# Animal Models of Narcolepsy: From Orexin Deficiency to Immune Mechanisms and Regenerative Therapies

**DOI:** 10.3390/cimb47110874

**Published:** 2025-10-22

**Authors:** Oscar Arias-Carrión, Emmanuel Ortega-Robles

**Affiliations:** 1 División de Neurociencias, Clínica, Instituto Nacional de Rehabilitación Luis Guillermo Ibarra Ibarra, Mexico City 14389, Mexico; edortega@cinvestav.mx; 2 Tecnologico de Monterrey, Escuela de Medicina y Ciencias de la Salud, Mexico City 14380, Mexico

**Keywords:** narcolepsy, orexin (Hypocretin), animal models, autoimmunity, regenerative therapy, gene therapy, OX2R agonists, translational neuroscience, precision medicine

## Abstract

Animal models have been pivotal in uncovering the orexin (hypocretin) system as the fulcrum of sleep–wake regulation and in shaping therapeutic discovery for narcolepsy. Early canine and murine models established that orexin loss underlies narcolepsy type 1, while conditional and receptor-specific manipulations refined mechanistic insight. However, current paradigms capture only fragments of the human phenotype, often exaggerating cataplexy and under-representing narcolepsy type 2. Here, we follow the evolution of narcolepsy modelling from classical knockout and receptor-deficient systems to immune-driven and cell-replacement models, identifying critical translational gaps and proposing strategies to bridge them. We highlight how immune-competent mouse lines, astrocyte-to-neuron reprogramming, and patient-derived hypothalamic organoids bridge pathogenic insight with therapeutic innovation. Integrating these advances with small-molecule OX2R agonists, gene therapy, and multi-omics-based patient stratification defines a roadmap for moving beyond symptomatic control. This review seeks to unify immune, cellular, and computational perspectives to guide the next generation of animal models toward the prevention, repair, and long-term cure of narcolepsy.

## 1. Introduction

Animal models remain indispensable in neuroscience, offering mechanistic clarity and acting as scaffolds for therapeutic innovation that human studies alone cannot provide [1,2]. In narcolepsy—a chronic vigilance-state disorder typified by excessive daytime sleepiness (EDS) and, in its classic form, cataplexy—such models have translated behavioural phenotypes to cellular and circuit mechanisms with profound impact [1,3]. The first formal clinical descriptions by Westphal (1877) [4] and Gélineau (1880) [5] distinguished narcolepsy from epilepsy on the basis of sudden, emotion-triggered loss of muscle tone in the presence of intact awareness.

For much of the 20th century, the diagnosis of narcolepsy rested on the “narcoleptic tetrad”: irresistible daytime sleep episodes, cataplexy, hypnagogic hallucinations, and sleep paralysis [3,6,7]. While cataplexy remains diagnostic of type 1 narcolepsy (NT1), the clinical heterogeneity—especially in type 2 narcolepsy (NT2) and pediatric populations—continues to challenge diagnostic certainty [8]. Insights from sleep science, including the delineation of distinct neurobiological substrates and the two-process model of sleep regulation, recast narcolepsy as fundamentally a disorder of REM regulation. Patients manifest not only EDS and disrupted nocturnal sleep but also sleep-onset REM periods (SOREMs)—a pathological intrusion of REM features into wakefulness [9].

This conceptual framing has evolved into a broader paradigm of arousal-state instability, in which the physiological boundaries separating wakefulness, non–REM, and REM states become increasingly blurred. Although patients retain homeostatic responses—such as rebound slow-wave activity after sleep deprivation—they often exhibit attenuated circadian amplitude and impaired arousal-promoting drive [10]. Diagnostic methods, such as the Multiple Sleep Latency Test (MSLT), continue to stratify a spectrum from NT1, characterized by cataplexy and orexin deficiency, to NT2, where orexin is preserved but instability in vigilance states predominates [11]. Nevertheless, diagnostic ambiguity persists, indicating that biomarker innovation and refined phenotyping remain urgent priorities [12].

Animal models have played a central role in elucidating the function of the orexin (hypocretin) system in regulating sleep–wake balance [1] (Table 1). Naturally occurring canine narcoleptics, bearing mutations in the orexin receptor 2 gene, first established a link between receptor dysfunction and cataplexy [13]. Knockout models (prepro-orexin null mice) and toxin-mediated ablation models (orexin/ataxin-3 transgenics) later confirmed that both peptide loss and neuronal cell death reproduce core narcoleptic features [14,15]. More sophisticated tools—optogenetic and chemogenetic control of orexin neurons, Cre-driven ablation, and immune-mediated ablation in Orexin-HA mice—now permit a causal dissection of sleep–wake microcircuits [10,16]. Nevertheless, no single model wholly emulates human narcolepsy: rodent systems often exaggerate cataplexy frequency, canine models display polyphasic sleep patterns, and none fully mirror the subtler NT2 phenotypes.

These experimental limitations parallel the major clinical unmet needs. Narcolepsy remains a lifelong disorder managed mainly through symptomatic treatments—wake-promoting agents, antidepressants, and oxybate formulations—that do not restore orexin neurons or halt disease progression [1,17]. Reliable biomarkers are still lacking for NT2 and pediatric forms, and the autoimmune basis of type 1 is incompletely modelled in current experimental systems. Therapeutic strategies derived from models focused on cataplexy may fail in real-world patients, where NT2 and mixed phenotypes are more prevalent. Addressing these gaps requires next-generation models that bridge immune and cellular approaches: immune-mediated models that recapitulate the proposed autoimmune aetiology of NT1 [11], partial-loss models mapping graded orexin deficiency to phenotypic spectra, and regenerative strategies—for example, astrocyte-to-neuron reprogramming [18] or iPSC-derived hypothalamic organoids [1,19]—to reconstitute or protect orexin signalling. Such steps chart a trajectory from symptomatic models to mechanistic tools and ultimately to disease-modifying interventions.

This review examines the evolution of animal models of narcolepsy, from receptor-deficient and knockout systems to immune-mediated and regenerative paradigms. These models represent complementary phases along a translational continuum: classical genetic and receptor-based systems established the mechanistic foundations by linking orexin deficiency to arousal-state instability, while immune and regenerative approaches extend this framework toward strategies capable of preventing or reversing neuronal loss. Their translational relevance is judged through construct, face, and predictive validity, considering how faithfully each model reproduces core mechanisms, mirrors human phenotypes, and anticipates therapeutic responses. Successful models also demonstrate in vivo indicators consistent with human narcolepsy—such as altered vigilance stability, REM–NREM dynamics, and cataplexy-like episodes—and exhibit pharmacological responsiveness comparable to that observed clinically with agents like modafinil, sodium oxybate, or OX2R agonists. Through comparative analysis across species and methodologies, we highlight both the strengths and limitations of current paradigms and propose directions for improving their clinical relevance. Bridging these gaps will refine our understanding of orexin-related mechanisms and accelerate the translation of preclinical discoveries into therapies that restore or protect arousal circuits, paving the way toward preventive and potentially curative interventions.

**Table 1:** Comparative overview of animal models of narcolepsy.

Model			Ref.	Genotype	Phenotype						Advantages and Disadvantages	Translational Potential and Limitations
					SWF	SOREMs	Wakefulness	REM time	Cataplexy	Obesity		
Canine	Spontaneous	Canarc-1	[20]	Mutation in *Hcrtr2* gene	+	+	↓		+		A: natural model; stable, fully penetrant phenotype; replicates spontaneous disease D: limited availability, ethical constraints, few molecular tools; cost and care; behavioural variability	P: strong face and construct validity; excellent model for cataplexy and receptor-based therapies L: limited scalability and genetic tractability restrict its routine use
Murine	Knockout	OX^−/−^	[21]	Prepro- orexin gene knockout	+	+	↓	↑	+	+	A: highly reproducible; well-characterized; compatible with pharmacological testing D: congenital loss may cause compensatory changes	P: high construct validity for total orexin deficiency; useful for drug screening L: lacks immune and progressive features of human disease
		OX/AT3	[15]	Selectively ablation of orexin cells through the expression of ataxin-3 transgene causes apoptosis	+	+	↓	↑	+	+	A: models time-dependent neuronal loss; consistent phenotype D: transgene expression irreversible; requires breeding colonies	P: mimics degenerative aspects of human narcolepsy; good predictive validity for restorative therapies L: onset earlier than in patients
		OX1R^−/−^	[22]	Single or double knockout for orexin receptors	+						A: dissects receptor-specific functions D: less severe phenotype in single KOs; redundancy between receptors complicates interpretation	P: useful for validating OX2R-selective agonists L: moderate face validity, as neuronal loss is absent; lacks progressive degeneration
		OX2R^−/−^	[14]		+				+			
		OX1R^−/−^/OX2R^−/−^	[23]		+	+	↓	↑	+			
		O/E3^−/−^	[24]	O/E3 transcription factor knockout	+	+	↓	↑	+		A: useful in studying the role of O/E3 in regulating sleep–wake controlling neurons D: embryonic lethality limits behavioral testing; broader developmental issues	P: reveals developmental mechanisms L: elucidates orexin lineage development rather than disease pathogenesis; low direct translational value
	Controlled	OX2R–TD	[25]	loxP-flanked transcription- disrupter gene cassette that prevents expression of OX2R	+		↓		+		A: reversible and controllable disruption of OX2R signaling; avoids developmental compensation seen in full knockouts D: requires complex breeding or viral delivery; phenotype milder than in complete receptor or peptide knockouts	P: enables testing of reversible loss of OX2R function and recovery; valuable for validating receptor-targeted drugs L: limited cataplexy and mild sleep–wake instability reduce its face validity compared to full OX2R^−^/^−^ or OX^−^/^−^ models
		OX-tTA TetO-DTA	[26]	Induction of diphtheria toxin A (DTA) in orexin neurons via tetracycline-transactivator system (tTA)	+	+	↓	↑	+	+	A: temporal control over cell loss; reversible induction; severe phenotype D: requires careful dosing and timing; complex to breed and manage; abrupt degeneration	P: models adult-onset loss with improved construct validity L: abrupt ablation limits chronic adaptation study
	Optogenetic	OX/HaloR	[27]	Expresses halorhodopsin (HaloR) in orexin neurons			↓				A: real-time control of neuronal activation or inhibition; causal circuit testing D: needs implantation of optical fibre; not a full narcolepsy phenotype; artificial activation patterns	P: powerful for dissecting network mechanisms L: limited predictive validity for chronic disease or pharmacotherapy
		OX/Arch	[28]	Expresses archaerhodopsin-3 (Arch) in orexin neurons	+		↓	↑		+		
		OX-tTA TetO-ArchT	[29]	Expresses ArchT using the tet-off (tTA) system	+		↓					
	Immune-driven	H1N1 infection	[30]	Orexin neuron ablation in Rag1^−/−^ mice through H1N1 infection	+	+	↓	↑			A: mimics autoimmune mechanisms D: complex to generate; limited immune system representation; incomplete phenotype expression	P: closest mimic of autoimmune etiology in NT1; highly informative for pathogenesis studies L: complex and variable immune response limits reproducibility
		OX-HA	[31]	Expresses hemagglutinin (HA) as a neo-self-antigen in orexin neurons					+			
Zebrafish (*Danio rerio*)		*hcrtr^168^*	[32]	Mutation in orexin/hypocretin receptor	+		↓				A: simple vertebrate model; optical accessibility; high-throughput screening D: lacks REM and cataplexy equivalents	P: conserved orexin pathway enables rapid drug discovery L: low face validity for mammalian narcolepsy
		Tg[*hcrt*:nfsB-EGFP]	[33]	Selective ablation of orexin neurons via nitroreductase–metronidazole treatment								
		p2ry11^−/−^	[34]	Loss-of-function *p2ry11* mutant showing reduced *hcrt* expression and orexin neuron loss								

Present (+); increased (↑); decreased (↓); sleep–wake fragmentation (SWF); sleep onset REM periods (SOREMs); advantages (A); disadvantages (D); potential (P); limitation (L).

## 2. Neurochemical Imbalances in Canine Narcolepsy: Insights from Pharmacology

Narcoleptic phenotypes have been documented across several mammalian species, including horses, cattle, sheep, and cats; however, the first systematically characterized case occurred in a Dachshund, which prompted William Dement to establish a colony of narcoleptic dogs at Stanford University. This resource encompassed multiple breeds, including Doberman Pinschers, Labrador Retrievers, Miniature Poodles, Dachshunds, Beagles, and Saint Bernards, providing a unique preclinical platform for mechanistic studies and therapeutic exploration [35]. Although narcoleptic manifestations were broadly conserved, marked differences in severity, age at onset, and disease progression were noted across breeds, highlighting the genetic and phenotypic heterogeneity of the disorder [36].

Polysomnographic investigations revealed that narcoleptic dogs retained largely normal baseline sleep architecture but displayed recurrent sleep-onset REM periods (SOREMs) and frequent cataplexy, often triggered by positive emotions such as play or food anticipation. Although the high baseline sleep requirements of dogs initially obscured recognition of excessive daytime sleepiness (EDS), systematic studies confirmed that narcoleptic animals exhibited increased drowsiness, reduced locomotor activity, fragmented sleep, and shortened sleep latencies under homeostatic challenge conditions [37].

Breeding experiments further clarified the heritability of canine narcolepsy. In large breeds, the familial form was mapped to an autosomal recessive locus, canarc-1, whereas inheritance in other breeds proved more complex. The establishment of a genetically stable colony at Stanford enabled systematic pharmacological, neurochemical, electrophysiological, and genetic investigations, providing an unparalleled resource for testing candidate therapies [35,38]. These studies converged on a model in which canine narcolepsy arises from brainstem neurochemical imbalances, characterized by reduced monoaminergic tone in concert with heightened cholinergic sensitivity. This framework advanced understanding of cataplexy pathophysiology and guided pharmacological interventions targeting neurotransmitter systems, including the refinement of stimulant and anticataplectic treatments [3].

## 3. Identifying the Genetic Defect in Canine Narcolepsy: Mutation in the Orexin-2 Receptor

In parallel, advances in human narcolepsy research have highlighted its strong association with the major histocompatibility complex (MHC) class II allele HLA-DR2, and subsequently with DQB1*06:02 and DQA1*01:02, alleles carried by the vast majority of patients compared to a minority of the general population [11]. These robust genetic associations supported an autoimmune basis for narcolepsy, suggesting selective immune-mediated destruction of orexin-producing neurons.

Canine research proved equally transformative. Although initial studies did not establish a link between canarc-1 and MHC class II loci, the creation of a bacterial artificial chromosome (BAC) library from a backcrossed Doberman pinscher heterozygous for canarc-1 ultimately identified a mutation in the orexin-2 receptor (OX2R) gene as the causal defect [13]. This discovery provided direct evidence that impaired orexin signalling alone is sufficient to generate narcoleptic phenotypes. More importantly, it consolidated the orexin (hypocretin) system as a fundamental regulator of sleep–wake stability, offering a molecular framework that bridged animal and human narcolepsy.

These insights underscore the enduring translational value of canine narcolepsy. By linking neurochemical imbalance with receptor-level defects, canine studies not only informed pharmacological approaches but also provided a conceptual template for immune and regenerative strategies now under investigation. As the field moves beyond symptom control, models that capture both the immune-mediated aetiology of NT1 and the subtler phenotypes of NT2 will be essential for driving a paradigm shift towards disease-modifying interventions.

## 4. Orexin and Narcolepsy

### 4.1. Orexin Receptors and Their Ligands: Molecular Foundations of Arousal Regulation

**Figure 1:**
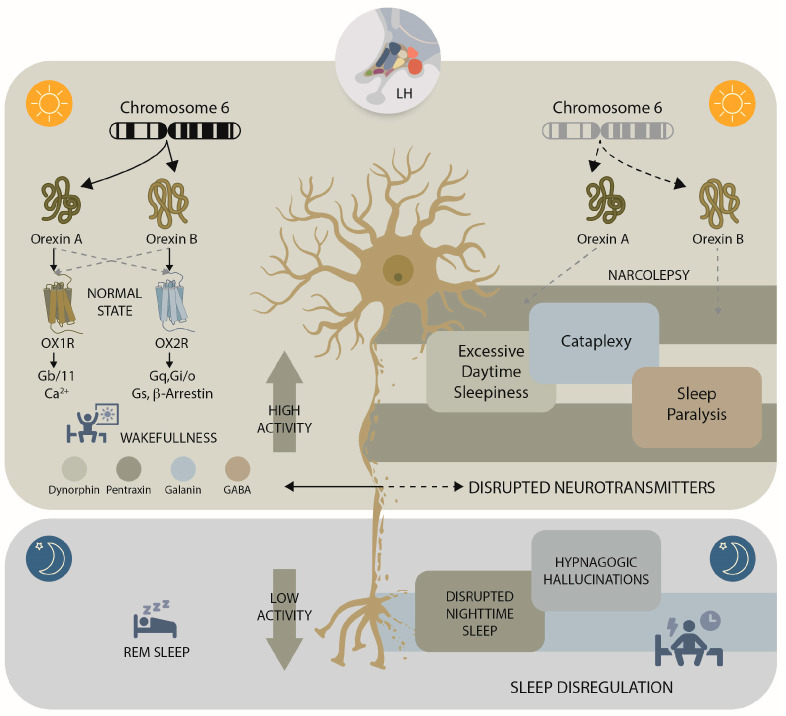
Physiological pathways of the orexin system in sleep–wake regulation and narcolepsy. Under physiological conditions, the *HCRT* gene on chromosome 6 encodes prepro-orexin in neurons of the lateral hypothalamus. Proteolytic processing generates orexin-A and orexin-B, which act on their receptors (OX1R and OX2R) to stabilize wakefulness. Orexin receptor activation engages downstream signalling cascades (Gq/11, Ca^2+^ influx, Gi/o, Gs, and β-arrestin pathways) and modulates co-transmitters including dynorphin, pentraxin, galanin, and GABA, thereby reinforcing excitatory drive to monoaminergic and cholinergic arousal centers. This coordinated network maintains consolidated wakefulness during the day and prevents the inappropriate intrusion of REM sleep into wakefulness. In narcolepsy (right panel), selective orexin deficiency or impaired receptor signalling disrupts these circuits, producing excessive daytime sleepiness, cataplexy, and sleep paralysis. Dysregulated neurotransmitter interactions drive abnormal state transitions, leading to nocturnal sleep fragmentation, disrupted REM sleep, hypnagogic hallucinations, and global instability of vigilance states.

The discovery of the orexin (hypocretin) system in 1998 marked a decisive turning point in sleep research. Using subtractive hybridization from rat hypothalamus, two previously unknown excitatory neuropeptides were identified: orexin-1 and orexin-2 (later termed orexin-A and orexin-B). Independently, another group demonstrated that these peptides act as endogenous ligands for two orphan G-protein–coupled receptors and stimulate feeding behaviour [39,40]. These receptors were named orexin receptor 1 (OX1R/HcrtR1), which displays preferential affinity for orexin-A, and orexin receptor 2 (OX2R/HcrtR2), which binds both peptides with comparable strength [41] (Figure 1).

Orexin neurons are localized within the perifornical hypothalamus and the lateral hypothalamic area, but they project widely across the central nervous system. Their axons innervate key arousal and reward centres, including the locus coeruleus, dorsal raphe nuclei, basal forebrain, amygdala, nucleus accumbens, and suprachiasmatic nucleus, as well as brainstem cholinergic nuclei and spinal cord circuits [42,43]. This extensive connectivity establishes orexin signalling as a master regulator of vigilance, stabilizing behavioural states and coordinating transitions between wakefulness, non-REM, and REM sleep.

### 4.2. Orexin Neurons as Integrators of Wakefulness Circuitry

The hypothalamus was implicated in arousal regulation almost a century ago, when von Economo observed that lesions in the posterior hypothalamus were associated with hypersomnolence in encephalitis lethargica. The identification of orexin neurons consolidated this link, positioning the hypothalamus as a central hub for sustaining wakefulness [44] (Figure 1).

Wake-promoting monoaminergic neurons in the brainstem contribute to cortical desynchronization and suppress REM-active pontine neurons, while cholinergic neurons of the basal forebrain and pons promote cortical activation during both wake and REM sleep [45]. Orexin neurons innervate and excite these populations, integrating arousal-promoting mechanisms into a coherent network [46]. Loss of orexin function destabilizes this system: canine narcolepsy models with mutations in OX2R show profound state instability, providing compelling evidence that orexin signalling is indispensable for maintaining consolidated wakefulness and orderly transitions across vigilance states [13,40].

### 4.3. Towards a Paradigm Shift

Understanding the orexin system has redefined narcolepsy as a disorder of arousal network instability, rather than merely a REM sleep dysregulation. This framework offers a powerful translational platform: while receptor mutations in canines illuminated the molecular basis of state fragmentation, recent human studies implicate immune-mediated loss of orexin neurons in narcolepsy type 1, with genetic and environmental factors modulating vulnerability [9,11]. Looking forward, therapies aimed at restoring orexin tone—through small molecules, gene therapy, or stem cell–derived hypothalamic transplants—hold the potential to transform narcolepsy from a chronic, symptomatic condition into a treatable or preventable disorder [1].

## 5. Murine Models of Narcolepsy

### 5.1. Sleep Attacks and Narcoleptic Phenotypes in Prepro-Orexin Knockout Mice

Soon after cloning of the orexin/hypocretin gene, deletion of prepro-orexin in mice yielded the first robust murine model of narcolepsy, establishing a genetic foothold for mechanistic inquiry [21]. EEG/EMG recordings later resolved initial behavioural arrests during the dark phase as narcolepsy-like features—sleep-onset REM periods (SOREMs), severe sleep–wake fragmentation, and increased sleepiness during the active period—together with gait ataxia [3,21]. These data showed that perturbation of a single neuropeptide system can reorganize vigilance states at scale, positioning the orexin system at the core of arousal stability [44].

Concurrently, naturally occurring canine narcolepsy was traced to mutations in the *Hcrtr2* gene. The cross-species convergence—presynaptic loss of orexin peptides in mice and postsynaptic OX2R dysfunction in dogs—established that impaired orexin signalling, at either node, is sufficient to produce narcoleptic phenotypes [13,14] (Figure 1).

### 5.2. Post-Mortem and Biomarker Evidence in Humans

Translation to the clinic accelerated once CSF orexin-A/hypocretin-1 testing showed undetectable or markedly reduced levels in most patients with NT1, enabling a biologically anchored distinction from NT2 [47,48]. Post-mortem studies confirmed a profound, selective loss of orexin neurons in NT1, with preservation of neighbouring melanin-concentrating hormone (MCH) neurons and gliosis in the orexin cell region [49,50]. Together, these data cemented orexin deficiency as the pathogenic substrate in human NT1 and defined a measurable biomarker (CSF orexin-A ≤ 110 pg/mL) with direct diagnostic utility [47,48].

### 5.3. The Orexin/Ataxin-3 (ATAX) Model: Neuronal Ablation and Disease Evolution

Because prepro-orexin knockout mice lack the peptides but retain orexin neurons, orexin/ataxin-3 (ATXN3) mice were created to ablate orexin neurons selectively. ATAX mice develop progressive degeneration, mirroring human disease evolution, and display the cardinal features of narcolepsy—fragmented sleep–wake cycles, SOREMs, obesity despite reduced intake, and cataplexy—with inter-individual variability in episode frequency and duration [15,26].

### 5.4. Receptor Genetics: Parsing Orexin Receptors (OX1R and OX2R) Contributions

Receptor-specific models clarify pathway anatomy. *Hcrtr1*/OX1R knockout mice show relatively mild abnormalities without frank cataplexy, whereas *Hcrtr2*/OX2R knockout mice exhibit more severe narcolepsy-like phenotypes, including cataplexy. Dual OX1R/OX2R knockouts exhibit robust cataplexy and SOREMs, underscoring the complementary roles of both receptors, with OX2R serving as the dominant mediator of sleep–wake stability [14,25]. These distinctions provide a mechanistic rationale for OX2R-selective agonists now in clinical development.

## 6. Other Rodent Models Informing Pathogenesis

### 6.1. Neuronal Transcription Factor O/E3 and Orexin Lineage Differentiation

The helix–loop–helix transcription factor O/E3 (EBF2) participates in hypothalamic neuronal differentiation and is expressed in orexin neurons. O/E3-null mice show marked loss of orexin neurons, disrupted orexinergic projections to sleep–wake nuclei, fragmented sleep, and SOREMs—phenotypes reversed by intracerebroventricular orexin-1, indicating that the sleep disorder arises from orexin-lineage failure rather than global developmental defects [24,51].

### 6.2. Autoimmunity, Human Leukocyte Antigen (HLA), and T-Cell Biology

Genetic studies consistently associate NT1 with HLA-DQB1*06:02 (and DQA1*01:02) and with polymorphisms in the T-cell receptor α (TRA) locus, suggesting a role in antigen presentation and T-cell surveillance [11]. Epidemiology following the 2009 H1N1 influenza pandemic—natural infection in China and exposure to the AS03-adjuvanted vaccine (Pandemrix) in parts of Europe—showed an increased incidence of narcolepsy, supporting the immune hypothesis [52,53]. Mechanistically, OX-HA mice—a model expressing hemagglutinin as a neo-self antigen in orexin neurons—develop narcolepsy-like phenotypes following adoptive transfer of antigen-specific CD8^+^ T cells, demonstrating that cytotoxic T cells can ablate orexin neurons in vivo [31]. In patients, autoreactive CD4^+^ and CD8^+^ T cells targeting orexin-neuron antigens have been identified, thereby consolidating a T cell–mediated pathogenesis in NT1 [54,55].

### 6.3. Distinct Yet Intertwined: Melanin-Concentrating Hormone and Orexin Neurons

Although anatomically intermingled, MCH and orexin neurons exert opposite influences on vigilance. Pharmacological and optogenetic studies demonstrate that MCH neurons promote REM (and variably NREM) sleep and facilitate NREM-to-REM transitions; conversely, inhibition or ablation can reduce REM stability or induce partial insomnia, with effects on hippocampus-dependent memory [27,29,56,57]. These data locate MCH neurons as modulators of REM dynamics and memory processing.

### 6.4. Dual Ablation of Orexin and Melanin-Concentrating Hormone Neurons

To probe circuit interactions, OXMC mice lacking both orexin and MCH neurons display more severe narcoleptic phenotypes—profound sleep fragmentation, increased cataplexy, and abnormal EEG signatures—than orexin-only ablations, implying that MCH pathways can buffer or shape symptom severity [58].

### 6.5. Translational Inflexion: From Models to Disease Modification

Receptor-level insights have catalyzed therapeutics that restore orexin tone. Intravenous danavorexton (TAK-925), an OX2R-selective agonist, improves objective and subjective sleepiness and vigilance across models and early human studies; oral oveporexton (TAK-861) has shown Phase 2 efficacy in NT1 with clinically meaningful reductions in EDS and cataplexy, marking a shift from symptomatic control to mechanistically targeted therapy [59,60]. Future directions include immunomodulatory strategies guided by T-cell biology and regenerative approaches (e.g., stem-cell–derived hypothalamic lineages) to reconstitute orexin signalling [44,61].

### 6.6. Challenges of IMMUNE-Mediated and Regenerative Models

While immune-mediated and regenerative models represent critical advances toward mechanistic fidelity and therapeutic innovation, their feasibility and reproducibility remain limited by methodological complexity. Immune paradigms, such as Orexin-HA or H1N1-sensitized mice, reproduce selective orexin-neuron loss but require precise genetic backgrounds and immune challenges that are difficult to standardize, leading to variable phenotypic penetrance across laboratories [51,62]. Similarly, regenerative approaches—including astrocyte-to-neuron reprogramming and transplantation of iPSC-derived hypothalamic neurons—demonstrate proof-of-concept restoration of hypocretin signaling but face major technical and ethical constraints [18,19]. These include the need for long-term graft integration studies, immunological compatibility testing, and adherence to ethical guidelines for stem-cell research and potential human–animal chimeras. Ensuring reproducibility will require standardized differentiation protocols, transparent reporting of cell-line provenance, and harmonized outcome metrics [63]. Immune and regenerative models, while promising, remain experimental tools whose translation to therapy demands require both technical refinement and rigorous ethical governance.

## 7. Non-Mammalian Models of Narcolepsy

To our knowledge, zebrafish (*Danio rerio*) represent the only established non-mammalian models of narcolepsy. Their conserved orexin system, optical transparency, and amenability to genetic manipulation make them uniquely suited for dissecting sleep–wake mechanisms at cellular resolution. As noted by AlAhmady and Alkhulaifi [63], zebrafish provide a scalable vertebrate platform for exploring orexin signaling and its behavioral correlates. The first receptor-deficient mutant, described by Yokogawa et al., carries a nonsense mutation in the single orexin receptor gene (*hcrtr^168^*), resulting in fragmented nocturnal sleep and reduced arousal [32]. Elbaz et al. later developed the inducible Tg[*hcrt*:nfsB-EGFP] line, in which metronidazole treatment selectively ablates orexin neurons, producing reversible sleep–wake fragmentation [33]. More recently, Zhao et al. created a CRISPR/Cas9 *p2ry11^−/−^* mutant linking purinergic signaling to orexin neuron survival; these fish display reduced *hcrt* expression, loss of orexin neurons, and excessive daytime sleepiness [34]. Although zebrafish lack REM physiology and cataplexy, these models collectively demonstrate conserved orexin-dependent control of arousal and extend narcolepsy research into immune and regenerative mechanisms.

## 8. Discussion

Animal models have been pivotal in establishing the orexin system as the fulcrum of sleep–wake stability, reproducing key features such as fragmented sleep, sleep-onset REM periods (SOREMs), cataplexy, and—in some paradigms—weight gain without hyperphagia [44,61]. Nevertheless, each model interrogates only a slice of the syndrome (Table 2). Optogenetic and chemogenetic tools afford millisecond precision over orexin neurons but risk reducing a chronic, degenerative disorder to a reversible “on–off” perturbation that lacks ecological validity [44,61]. In contrast, neuron-ablation paradigms (for example, orexin/ataxin-3 or conditional diphtheria-toxin systems) better mirror the progressive loss and its network consequences, although degeneration is still driven by artificial temporal control and constrained by species-specific sleep architecture [15,26,58].

Translational blind spots persist. Canine models have historically been transformative, but their use is complicated by polyphasic sleep, which limits direct comparison to human nocturnal consolidation. Rodents, with intrinsically fragmented sleep, present similar challenges. Most importantly, preclinical readouts often over-privilege cataplexy. While cataplexy is pathognomonic for NT1, it is uncommon across the day in many NT1 patients and absent in NT2. Overreliance on cataplexy as the principal endpoint risks skewing therapeutic discovery toward a single, exaggerated phenotype rather than the broader clinical spectrum that includes excessive daytime sleepiness, vigilance instability, and cognitive or autonomic burden [6].

A conspicuous gap is the absence of models that faithfully recapitulate NT2. Clinically, NT2 is defined by excessive daytime sleepiness with typical SOREMs but no cataplexy; nocturnal sleep is often relatively preserved, and REM latencies are longer than in NT1. Biomarker and post-mortem evidence suggest that NT2 reflects partial orexin dysfunction rather than the near-complete neuronal loss characteristic of NT1 [64]. Most available animal models—including receptor knockouts, peptide-null mutants, and full orexin-neuron ablation lines—closely mimic NT1 but fail to capture the subtler neurophysiological and behavioral profile of NT2 [51,62]. Recent studies in partially ablated OX-tTA;TetO-DTA mice and in strains with reversible or incomplete orexin suppression have begun to approximate this intermediate phenotype, supporting the view that NT1 and NT2 lie along a continuum of orexin deficiency. Clarifying this distinction has important diagnostic implications, as CSF orexin levels, SOREM frequency, and cataplexy expression remain key markers for differential diagnosis. Purpose-built models that titrate orexin tone to intermediate levels and assess outcomes beyond cataplexy—such as sleep–wake stability, REM propensity, and autonomic regulation—are therefore essential to extend therapeutic discovery across the full disease spectrum [3,64].

Insights from receptor-specific models have been particularly influential in shaping therapeutic innovation. OX1R- and OX2R-knockout mice revealed distinct contributions of each receptor subtype: OX2R loss produces profound sleep–wake fragmentation and cataplexy, whereas OX1R deletion yields milder phenotypes without cataplexy. This receptor dissociation guided the development of OX2R-selective agonists, such as danavorexton and oveporexton [59,60], which restore wakefulness and suppress cataplexy by targeting the principal pathway mediating sleep stability. In contrast, immune-mediated models—including H1N1-infected or Orexin-HA mice—have illuminated the autoimmune mechanisms leading to selective orexin-neuron loss, providing a foundation for future immunomodulatory interventions aimed at disease modification or prevention. Cell-replacement and reprogramming models demonstrate proof-of-concept for orexin restoration therapies, establishing a mechanistic bridge between neuronal loss and functional recovery.

Recent studies have further strengthened the evidence that narcolepsy type 1 is a T cell–mediated autoimmune disease targeting hypocretin neurons. The strong association with HLA-DQB1*06:02, supported by T-cell receptor polymorphisms, points to a genetically restricted immune response that underlies selective neuronal vulnerability [11]. Autoreactive CD4^+^ and CD8^+^ T cells recognizing hypocretin-related antigens have been demonstrated in patients, and adoptive transfer of antigen-specific T cells reproduces hypocretin-neuron loss and narcolepsy-like features in Orexin-HA mice [1,62]. These findings place adaptive immunity at the center of disease pathogenesis and provide a mechanistic link between environmental triggers such as H1N1 infection and genetically determined immune recognition [52,53]. Immune-competent animal models therefore represent a critical platform for testing preventive or immunomodulatory strategies, including regulatory T-cell activation, peptide-based tolerization, and checkpoint modulation.

Although animal models have been indispensable for dissecting orexin circuitry and testing emerging therapies, several methodological and biological factors constrain their translational applicability. A major limitation is the overemphasis on cataplexy as the primary behavioral endpoint. While cataplexy is pathognomonic for NT1, its exaggerated frequency in many animal models and absence in NT2 restrict its value as a universal metric [62]. Incorporating quantitative behavioral and electrophysiological measures—such as vigilance stability, frequency of sleep–wake transitions, EEG/EMG spectral analyses of REM intrusions, latency-based indices (e.g., mean sleep latency or SOREM probability), and autonomic markers like heart-rate variability—would provide a more balanced and objective assessment of narcolepsy phenotypes. In parallel, sex differences remain underexplored, as most studies rely on male rodents despite evidence that hormonal modulation affects sleep–wake patterns and orexin signaling [65,66]. Circadian factors also merit consideration, since experimental timing and light–dark cycles can markedly influence neuronal activity, cataplexy frequency, and expression of sleep-related genes [67]. Moreover, interspecies variability in sleep architecture, orexin neuron distribution, and receptor affinity complicates direct extrapolation to humans [62]. Addressing these biological and methodological sources of variability through balanced study design, standardized recording parameters, and cross-species validation will be essential for improving the reproducibility and translational precision of future narcolepsy models.

Beyond their mechanistic value, animal models hold significant clinical relevance. Narcolepsy imposes a considerable psychosocial and functional burden, affecting learning, occupational performance, and driving safety, with increased risk of metabolic, psychiatric, and cardiovascular comorbidities [3,6]. Bridging preclinical insights with these clinical realities underscores the urgency of developing disease-modifying interventions. Immune and regenerative models, by clarifying how hypocretin neurons are lost and how they might be replaced or protected, directly inform the development of targeted therapies that could restore normal sleep–wake regulation and improve quality of life.

Contemporary management remains symptomatic mainly. Wake-promoting agents, anticonvulsant therapies, and oxybate formulations improve function but do not address the loss of orexin neurons. Two converging avenues now enable a mechanistic pivot. First, OX2R-selective agonists restore downstream signalling independent of cell replacement. Intravenous danavorexton (TAK-925) produced rapid wake promotion in translational studies and early human trials [59], and the oral agonist oveporexton (TAK-861) yielded Phase 2 improvements in wakefulness and cataplexy in NT1, signalling a shift from symptomatic stimulation to circuit-specific rescue [60]. Second, regenerative and circuit-repair strategies—including stem-cell–derived hypothalamic lineages and glial reprogramming—are advancing preclinically and could, in time, rebuild orexin tone rather than merely pharmacologically emulate it [1].

Convergent genetic, epidemiologic, and cellular data support a T-cell–mediated pathogenesis in NT1, anchored by HLA-DQB1*06:02 and T-cell receptor associations, with infection or vaccination as modulators of risk [11,53]. Human studies have identified autoreactive CD4^+^ and CD8^+^ T cells targeting orexin-neuron antigens [54,55]. Causality has been modelled directly: adoptive transfer of antigen-specific CD8^+^ T cells ablates orexin neurons and induces a narcolepsy-like phenotype in Orexin-HA mice [31]. Immune-relevant models should now be mainstreamed to dissect the cascade from genetic susceptibility to neuronal loss, identify windows for prevention, and test immunomodulatory interventions alongside receptor agonism.

Judging the success of narcolepsy models requires assessing how well they reproduce both the observable phenotype and the underlying mechanisms of disease. Mechanistic fidelity remains central—models that replicate hypocretin loss, receptor dysfunction, or immune-mediated neuronal injury offer the strongest construct validity. Yet molecular or histological confirmation alone is insufficient; true translational success must also be reflected in measurable outcomes in living animals. Mechanistic disruptions should manifest as appearance indicators such as altered vigilance stability, excessive daytime sleepiness, cataplexy-like episodes, and changes in sleep architecture. Additional quantitative measures—including EEG/EMG spectral dynamics, sleep-onset latency, locomotor activity, and heart-rate variability—provide objective readouts of arousal-state instability and bridge mechanism with phenotype. Equally important is predictive capacity—the ability of models to anticipate therapeutic responses to pharmacological, immunomodulatory, or regenerative interventions, with consistency between drug effects in animals and clinical outcomes (e.g., the wake-promoting actions of modafinil, sodium oxybate, or OX2R agonists) serving as a key benchmark of validity. Ultimately, the most valuable models integrate mechanistic accuracy, in vivo reproducibility, and pharmacological predictability, providing robust platforms for testing interventions that preserve or restore arousal network integrity.

Building on these principles, future progress will depend on aligning models with the heterogeneity of human disease. NT2-focused paradigms, immune-competent models that capture T-cell effector mechanisms, and partial-loss systems calibrated to intermediate orexin deficits are priorities. Equally, preclinical endpoints must expand beyond cataplexy to include objective wakefulness, vigilance stability, cognition, and autonomic outcomes—mirroring patient-centred clinical trials [59,60]. Together, these steps will transform animal systems from partial mirrors into robust translational platforms, accelerating a shift from symptomatic management to disease-modifying or preventive therapy.

## 9. Conclusions

Animal models have been indispensable in establishing the orexin/hypocretin system as a central regulator of sleep–wake stability and in clarifying the pathophysiology of narcolepsy. From canine studies that revealed receptor mutations to murine models that demonstrated peptide loss and neuronal degeneration, these systems have progressively shaped our mechanistic understanding. More recent immune-mediated and regenerative paradigms now highlight the complexity of narcolepsy type 1 as an autoimmune disorder and underscore the need for models that faithfully capture narcolepsy type 2, where orexin function is only partially impaired.

Despite their limitations, preclinical models continue to guide therapeutic discovery. OX2R-selective agonists, gene therapy approaches, and regenerative strategies are beginning to move the field beyond symptomatic relief toward disease modification. Parallel integration of immune-relevant models, patient-derived hypothalamic organoids, and multi-omics technologies offers a path to refine patient stratification and to identify opportunities for prevention.

Nevertheless, important research gaps remain. Most models overrepresent narcolepsy type 1, whereas type 2 and pediatric variants are poorly captured. Immune-mediated paradigms lack reproducibility and mechanistic resolution, and regenerative models require validation of graft integration, long-term stability, and ethical oversight. Future progress will depend on several methodological priorities: developing partial-loss and immune-competent models that reflect the full clinical spectrum; standardizing behavioral and electrophysiological metrics beyond cataplexy frequency; incorporating sex, circadian, and interspecies factors into experimental designs; and integrating molecular, electrophysiological, and imaging endpoints to enhance cross-species translation.

By bridging immune and regenerative approaches and expanding methodological rigor, narcolepsy research is poised to transition from descriptive modeling to predictive and reparative frameworks—advancing toward precision medicine and the long-term goal of transforming a chronic symptomatic disorder into a preventable and potentially curable condition.

## Figures and Tables

**Table 2:** A roadmap for narcolepsy research.

Models and Mechanisms	Therapies and Future Directions
**Genetic and neuronal models**—including prepro-orexin knockouts, orexin/ataxin-3 ablations, receptor mutants, and canine *Hcrtr2* lines—have redefined narcolepsy as a disorder of orexin deficiency. These models provided face validity and mechanistic depth but remain locked in descriptive paradigms.	**Small-molecule OX2R agonists**—Danavorexton and oveporexton deliver the first truly mechanism-targeted therapies in sleep medicine, improving vigilance and reducing cataplexy. They signal a pivot from symptom management toward disease modification.
**Immune relevance**—Human HLA signatures, autoreactive T cells, and Orexin-HA mice prove that adaptive immunity can selectively erase orexin neurons. This finding reframes narcolepsy type 1 (NT1) as an autoimmune encephalopathy, placing it within the broader landscape of organ-specific autoimmunity.	**Gene therapy**—Viral orexin delivery or receptor reconstitution offers the possibility of durable circuit repair, with narcolepsy positioned to become the first neuropsychiatric disease amenable to one-time molecular correction.
**Circuit tools**, including optogenetics, chemogenetics, and conditional ablations, have mapped the causal architecture of sleep–wake transitions. Nevertheless, their artificial timing and reversibility risk trivialize a degenerative, chronic disorder into an on–off switch.	**Cellular therapies**—including stem-cell–derived hypothalamic neurons, astrocyte-to-neuron reprogramming, and patient-specific organoids—foreshadow a regenerative neurology, where lost arousal circuits are rebuilt rather than merely pharmacologically bypassed.
**Phenotypic blind spots**—Current models exaggerate cataplexy and under-represent narcolepsy type 2 (NT2), where partial orexin dysfunction, vigilance instability, and cognitive–autonomic burden dominate. Without NT2-specific paradigms, therapeutic discovery risks serving only a fraction of patients.	**Integrative approaches**—including multi-omics, systems biology, and AI-driven digital phenotyping—promise to bridge the gap between laboratory models and real-world heterogeneity, stratify patients, define novel biomarkers, and accelerate precision trials.
**Trajectory**—The field must break from its descriptive past. From genetic models anchoring orexin deficiency → to mechanistic circuit dissection → to immune-mediated causality → to regenerative and precision interventions.	**Central Goal**—To shift narcolepsy from a “managed” chronic disorder into one of the first preventable and curable neuropsychiatric conditions—a test case for how neuroscience can move from symptom palliation to circuit restoration and disease prevention.

## Data Availability

No new data were created or analyzed in this study. Data sharing is not applicable to this article.

## Abbreviations

The following abbreviations are used in this manuscript:

CSF	Cerebrospinal fluid
EEG	Electroencephalography
EMG	Electromyography
HLA	Human Leukocyte Antigen
REM	Rapid Eye Movement
NT1	Narcolepsy Type 1
NT2	Narcolepsy Type 2
MCH	Melanin-Concentrating Hormone
EDS	Excessive Daytime Sleepiness
CD8	Cluster of Differentiation 8 (cytotoxic T cell subset)
